# Decomposition and Comparative Analysis of Urban-Rural Disparities in eHealth Literacy Among Chinese University Students: Cross-Sectional Study

**DOI:** 10.2196/63671

**Published:** 2025-03-26

**Authors:** Yao Yu, Zhenning Liang, Qingping Zhou, Yusupujiang Tuersun, Siyuan Liu, Chenxi Wang, Yuying Xie, Xinyu Wang, Zhuotong Wu, Yi Qian

**Affiliations:** 1 School of Health Management Southern Medical University Guangzhou China; 2 The Seventh Affiliated Hospital Sun Yat-sen University Shenzhen China; 3 School of Public Health Southern Medical University Guangzhou China; 4 Shenzhen Longhua Maternity and Child Health Hospital Shenzhen China

**Keywords:** university students, eHealth literacy, urban-rural disparities, Fairlie decomposition model, health equity

## Abstract

**Background:**

Mobile health care is rapidly expanding in China, making the enhancement of eHealth literacy a crucial strategy for improving public health. However, the persistent urban-rural divide may contribute to disparities in eHealth literacy between urban and rural university students, potentially affecting their health-related behaviors and outcomes.

**Objective:**

This study aims to examine disparities in eHealth literacy between university students in urban and rural China, identifying key influencing factors and their contributions. The findings will help bridge these gaps, promote social equity, enhance overall health and well-being, and inform future advancements in the digital health era.

**Methods:**

The eHealth Literacy Scale (eHEALS) was used to assess eHealth literacy levels among 7230 university students from diverse schools and majors across 10 regions, including Guangdong Province, Shanghai Municipality, and Jiangsu Province. Descriptive statistics summarized demographic, sociological, and lifestyle characteristics. Chi-square tests examined the distribution of eHealth literacy between urban and rural students. A binary logistic regression model identified key influencing factors, while a Fairlie decomposition model quantified their contributions to the observed disparities.

**Results:**

The average eHealth literacy score among Chinese university students was 29.22 (SD 6.68), with 4135 out of 7230 (57.19%) scoring below the passing mark. Rural students had a significantly higher proportion of inadequate eHealth literacy (2837/4510, 62.90%) compared with urban students (1298/2720, 47.72%; P<.001). The Fairlie decomposition analysis showed that 71.4% of the disparity in eHealth literacy was attributable to urban-rural factors and unobserved variables, while 28.6% resulted from observed factors. The primary contributors were monthly per capita household income (13.4%), exercise habits (11.7%), and 9-item Patient Health Questionnaire (PHQ-9) scores (2.1%).

**Conclusions:**

Rural university students exhibit lower eHealth literacy levels than their urban counterparts, a disparity influenced by differences in socioeconomic status, individual lifestyles, and personal health status. These findings highlight the need for targeted intervention strategies, including (1) improving access to eHealth resources in rural and underserved areas; (2) fostering an environment that encourages physical activity to promote healthy behaviors; (3) expanding school-based mental health services to enhance health information processing capacity; and (4) implementing systematic eHealth literacy training with ongoing evaluation. These strategies will support equitable access to and utilization of eHealth resources for all students, regardless of their geographic location.

## Introduction

Health literacy is a midstream determinant of health that can improve outcomes, reduce inequalities, and promote health-related behaviors [1]. Today, the internet is widely used due to its accessibility, broad information coverage, ease of use, affordability, and anonymity [2]. It has become a key platform for disseminating health information, particularly in middle- and high-income countries such as China, where health care resources and face-to-face medical appointments are limited [3]. However, the widespread availability of the internet has also exposed shortcomings in online health information, especially concerning its quality [4]. Individuals frequently encounter false or dubious health information online, a challenge that is not new [5]. eHealth literacy extends the concept of health literacy into the digital domain. It was first introduced by Norman and Skinner [6]. Gilstad [7] later expanded its definition to encompass the ability to address health problems by communicating, locating, understanding, evaluating, and applying health information and digital technologies within specific cultural and social contexts [8]. It should be emphasized that eHealth literacy requires not only the ability to access necessary information but also the ability to assess the quality of eHealth information and distinguish between reliable and unreliable sources [6]. eHealth literacy is essential for maintaining good health and preventing disease. Improving eHealth literacy enables individuals to access health information for disease prevention, treatment, and health promotion [9]. People with strong eHealth literacy can make informed decisions, select appropriate health services, and manage their health effectively. This includes monitoring their health status, following medical advice, and taking preventive measures against disease. Furthermore, eHealth literacy can enhance communication between patients and physicians, improving the effectiveness and overall satisfaction of medical services [10].

As the future builders and pillars of development in their home countries, the health status of young people and their ability to maintain it deserve attention. Their knowledge, attitudes, and behaviors not only impact their own lives but also influence society as a whole [11,12]. For university students transitioning to adulthood, the development of health behaviors during this stage is crucial for maintaining a healthy lifestyle throughout adulthood [13,14]. However, they are exposed to various health risks [15-17]. At the same time, even students in good health often engage in risky health behaviors [18]. This highlights an increased need for reliable health information. Conducting an internet search may align with young people’s desire for autonomy and self-sufficiency [19]. Currently, it is estimated that most university students (99%) rely on the internet as their primary source of health-related information [20]. The eHealth Literacy Scale (eHEALS) has emerged as a valid measurement and intervention tool for promoting healthy behaviors among adolescents [21]. Improving eHealth literacy can encourage students to adopt healthier behaviors [22], better manage their health, and maintain both their physical and mental well-being [23,24]. However, despite frequently using digital media to access health information, a significant proportion of university students lack adequate eHealth literacy [25,26], which severely hinders disease prevention and the effectiveness of health services [27]. Stellefson et al [9] demonstrated that while students are confident in their ability to use the internet, their practical eHealth literacy is often insufficient. Moreover, another study found that a considerable proportion of students struggle to critically evaluate the health information they encounter online [26].

According to the latest data from the Chinese Ministry of Education, the total number of students enrolled in various forms of higher education in China in 2023 was 47.63 million [28]. Research on university students’ eHealth literacy is both important and urgent, particularly given the lack of a strict verification mechanism for health information on most websites in China [29]. While research on health literacy in China began relatively late, the widespread use of internet technology in health care has gradually brought eHealth literacy into public focus. As university students are a key demographic of internet users, they have become a focal point of eHealth literacy research due to their frequent use of digital tools and ability to access information. One major area of research is the localized adaptation of eHealth literacy measurement tools. In 2013, Guo et al [30] pioneered the translation and adaptation of eHEALS in China, focusing on adolescents, with a reported Cronbach alpha coefficient of 0.913. Subsequent applications of this version among university students have consistently demonstrated high reliability and validity [31,32]. Another important research direction is examining the current state of students’ eHealth literacy and analyzing the factors that influence it. Overall, the eHealth literacy of Chinese university students appears to be at a relatively optimistic level. In recent years, studies have shown that the average eHealth literacy scores of university students typically range between 27 and 29 points [33-35], with variations influenced by the time and region of the research. The key factors affecting university students’ eHealth literacy primarily include individual characteristics such as gender, major, academic performance, and place of origin (urban or rural). Family-related factors, such as income and number of siblings, as well as the students’ own health status, also play a significant role [36]. With the promotion of initiatives such as the “Healthy China Strategy” and the “14th Five-Year Plan for National Health Informatization,” the eHealth literacy of university students has become a key issue, highlighting the urgent need for intervention research. However, existing studies are largely limited to single-region samples, lacking large-scale cross-sectional surveys that cover multiple regions. Additionally, research on the determinants of eHealth literacy remains insufficient, with limited detailed data analysis and stratified comparisons among students with different characteristics. Furthermore, there is a lack of systematic comparisons on how variations in eHealth literacy impact health outcomes, making it difficult to establish a concrete and reliable empirical foundation for intervention research and policy development.

Existing academic research on the role of the internet in health communication and promotion often emphasizes macro-level social inequalities, commonly referred to as the digital divide [37,38]. eHealth literacy plays a crucial role in promoting health equity, improving health outcomes, and reducing health care costs [5], offering significant potential to bridge this divide. This study highlights the widening digital gap between urban and rural students in China, with a particular focus on the challenges faced by rural students as a digitally disadvantaged group. Specifically, it explores the following: (1) Are there differences in eHealth literacy between urban and rural students? (2) What similarities or differences exist in the factors influencing their eHealth literacy? (3) How do these factors contribute to the observed differences? The study’s results will serve as a reference for developing intervention methods and improvement strategies for eHealth literacy among students. Additionally, it seeks to recognize and address these social inequalities, ensuring that eHealth can truly benefit people worldwide, including those in marginalized and underserved areas.

## Methods

### Data Source

This study is a cross-sectional survey. From January to February 2023, the researcher distributed an anonymous electronic questionnaire via the Questionnaire Star platform [39] using a convenience sampling method. The questionnaire was distributed to university students across 10 regions, including Guangdong Province, Shanghai Municipality, and Jiangsu Province, covering majors such as economics, medicine, management, and literature.

**Figure 1:**
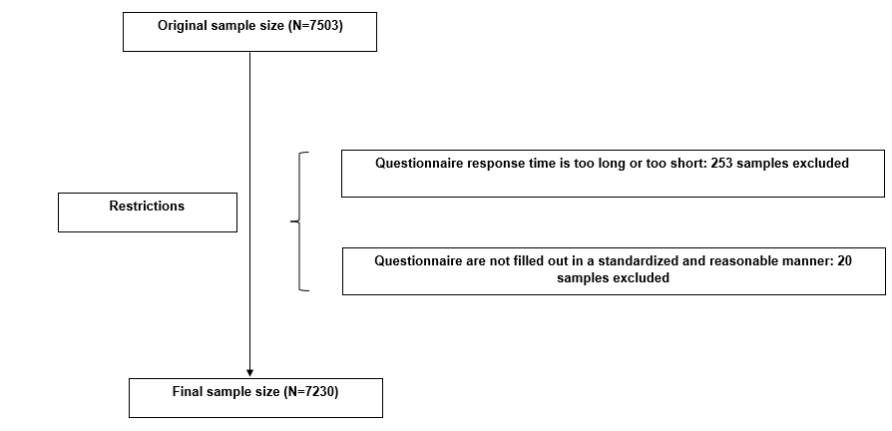
Participant screening flowchart.

To ensure data accuracy and reliability, the questionnaire included 2 verification questions: a repetitive question and a general knowledge question (“What is the capital of China?”). If a respondent answered either question incorrectly, the questionnaire was deemed invalid and excluded from further analysis. A total of 7503 questionnaires were collected. After screening, 253 questionnaires with anomalous response times and 20 with irregular completion patterns were excluded, resulting in 7230 valid questionnaires and an effective response rate of 96.36%. Participants in this study were required to meet the following inclusion criteria: (1) undergraduate students and (2) normal cognitive function with the ability to complete the questionnaire independently. The exclusion criterion was an incorrect response to the verification questions. The exclusion process is illustrated in Figure 1.

### eHealth Literacy

This study used the Chinese version of the eHEALS to assess university students’ eHealth literacy levels. The eHEALS was developed by Norman et al [6] in 2006, based on the concept of eHealth literacy and Lily’s model for evaluating an individual’s ability to search for, screen, understand, assess, and utilize health information [6]. Since its inception, eHEALS has become the most widely used scale for measuring eHealth literacy across different populations in contemporary studies [40]. The Cronbach alpha coefficient for eHEALS is high across numerous studies involving diverse populations, typically ranging from 0.84 to 0.94. This indicates that the scale demonstrates strong internal consistency and reliability. The scale consists of 8 items, categorized into 3 competency areas related to the use of online health information and services: application competencies (items 1-5), judgmental competencies (items 6 and 7), and decision-making competencies (item 8). The scale uses a 5-point Likert scale, with scores ranging from 1 (very poorly met) to 5 (very well met). The total score for the 8 items ranges from 8 to 40, with higher scores indicating a higher level of eHealth literacy. In this study, the widely recognized cut-off score of 32 was used to classify the eHealth literacy of urban and rural university students into 2 categories: “qualified” (≥32 points) and “unqualified” (<32 points) [33,41]. The overall Cronbach α coefficient for this study was 0.974, with 0.973 for the rural university student group and 0.974 for the urban university student group.

### Ethics Approval

The study was approved by the Biomedical Ethics Committee of Southern Medical University (Ethics Review Board [2023] No. 46). All participants signed an informed consent form before the survey began. The distribution, collection, and storage of questionnaires were managed via the Questionnaire Star platform. Data access was restricted to the researcher, and the information was tamper-proof to ensure security and integrity. This study strictly adhered to relevant laws and regulations. To protect participant privacy, all public reports will exclude personal identifiers. The final data set was exported in EpiData format for subsequent statistical analysis.

### Grouping Variables

Respondents were classified as rural or urban based on the household type they reported in the survey.

### Covariates

To obtain more reliable results, a range of potential confounding variables were controlled for, including demographic and sociological characteristics, personal lifestyles, and mental health status—factors previously considered in studies on eHealth literacy levels.

### Demographic Characteristics

Participants were classified based on 2 variables: gender (male or female) and personal religious belief (yes or no).

### Sociological Characteristics

Respondents were classified into 4 groups based on their academic performance, determined by class ranking: below 25%, 25%-50%, 50%-75%, and above 75%. Additionally, monthly per capita household income was categorized into 4 groups: less than 2500 RMB (1 RMB=US $0.14), 2500-<5000 RMB, 5000-10,000 RMB, and above 10,000 RMB.

### Personal Lifestyle

The variables of exercise, smoking, and alcohol use were dichotomized into a binary format, with responses categorized as “yes” or “no.” “Exercise” was defined as engaging in physical activity at least three times in the past month, with each session lasting a minimum of 30 minutes. Additionally, the use of tobacco products and alcoholic beverages (including distilled spirits, beer, wine, and rice wine) in the past month was recorded as a dichotomous variable (yes or no).

### Mental Health Status

The 7-item Generalized Anxiety Disorder (GAD-7) tool was used to assess the prevalence and severity of anxiety symptoms among study participants. This tool consists of 7 items, each rated on a 4-point scale ranging from 0 to 3: 0=“not at all,” 1=“a few days,” 2=“more than half the time,” and 3=“almost every day.” A score below 5 indicates the absence of anxiety symptoms, while a score of 5 or higher suggests the presence of anxiety symptoms [42]. In this study, the GAD-7 demonstrated a Cronbach α coefficient of 0.944.

The 9-item Patient Health Questionnaire (PHQ-9) was used to assess depressive symptoms among participants. This tool consists of 9 items and follows the same scoring method as the GAD-7. A score below 5 indicates the absence of depressive symptoms, whereas a score of 5 or higher suggests their presence [43]. In this study, the PHQ-9 demonstrated a Cronbach α coefficient of 0.931.

### Statistical Analysis

The demographic characteristics and lifestyles of the study population were analyzed descriptively. Continuous variables were presented as mean (SD), while categorical variables were expressed as percentages. A chi-square test was used to examine the distribution of eHealth literacy levels among urban and rural university students. Subsequently, a binary logistic regression model was constructed to identify key determinants of eHealth literacy levels in these groups. The outcome variable was defined as a binary classification of eHealth literacy (qualified/unqualified), while independent variables were selected from 4 domains: demographic characteristics, sociological characteristics, personal lifestyle, and mental health status. Initially, variables were screened using univariate analysis (retention criterion: *P*<.05) and subsequently included in the multivariate model. Statistically significant predictors (*P*<.05) were retained in the final model. All statistical analyses were performed using SPSS Statistics 23 (IBM Corp.). To examine the factors contributing to discrepancies in eHealth literacy levels between urban and rural university students, the Fairley model was applied using Stata MP 18.0 (StataCorp). The significance level was set at .05.

### Fairlie Decomposition Model

In this study, we applied a multivariate Fairlie decomposition analysis (FDA) based on a binary regression model. FDA is a decomposition technique used in multivariate models to quantify the contribution of predicted differences between 2 groups to an outcome variable. It extends the Blinder-Oaxaca decomposition, which has been widely criticized for its inefficiency in handling logit and probit models. FDA was specifically developed for nonlinear regression models, including logit and probit models [44]. The FDA quantifies the contribution of independent variables to differences between groups by calculating the change in the mean predicted probability when substituting 1 independent variable at a time in 1 group (eg, rural university students) while holding other variables constant in the other group (eg, urban university students). The Fairlie decomposition technique ensures that the predicted probability remains within the range of 0-1. Studies have shown that the FDA provides a more precise quantification of variable contributions and significance levels in nonlinear regression models [45-47]. Given that the dependent variable was dichotomous, we applied Fairlie nonlinear decomposition to attribute differences in university students’ eHealth literacy to various contributing factors. As outlined by Fairlie [48], the decomposition of the nonlinear equation can be expressed as follows:



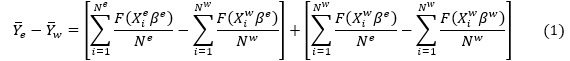



The symbols 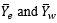 represent the mean probabilities of the 2 binary outcomes. The *F* is used to indicate the cumulative distribution function of the logistic distribution. *Y_e_*-*Y_w_* represents the total difference in the differences between the 2 groups; *N^e^* and *N^w^* represent the sample sizes of the 2 samples. In equation 1, the first term in parentheses represents the portion of the gap attributable to differences in observed characteristics, while the second term denotes the portion of the gap explained by differences in estimated coefficients.

## Results

### Basic Characteristics of the Study Participants

The study included a total of 7230 participants. The mean eHealth literacy score among university students was 29.22 (SD 6.68; Table 1). Prior research on eHealth literacy among Chinese university students has established a passing threshold of 32 points [33], with scores below this cutoff indicating insufficient eHealth literacy. As shown in Table 2, 4135 out of 7230 (57.19%) students did not meet the eHealth literacy standard, while 3095 out of 7230 (42.81%) students qualified. Notably, a higher proportion of rural university students (2837/4510, 62.90%) failed to meet the standard compared with their urban counterparts (1298/2720, 47.72%; *P*<.001).

The chi-square test results in Table 2 indicate significant differences in the distribution of 8 covariates between urban and rural university students. These covariates include gender (*P*<.001), religion (*P*<.001), class ranking (*P*=.02), monthly per capita household income (*P*<.001), exercise (*P*<.001), drinking (*P*<.001), GAD-7 scores (*P*=.002), and PHQ-9 scores (*P*=.04). However, no significant difference was observed in the distribution of smoking (*P*=.93).

**Table 1:** eHealth literacy score for university students.

Item	Overall, mean (SD)	Rural, mean (SD)	Urban, mean (SD)
Q1: I know how to find helpful health resources on the internet	3.69 (0.93)	3.59 (0.91)	3.86 (0.94)
Q2: I know how to use the internet to answer my health questions	3.67 (0.92)	3.57 (0.90)	3.85 (0.93)
Q3: I know what health resources are available on the internet	3.64 (0.91)	3.53 (0.89)	3.82 (0.91)
Q4: I know where to find helpful health resources on the internet	3.65 (0.90)	3.54 (0.88)	3.83 (0.91)
Q5: I know how to use the health information I find on the internet to help me	3.69 (0.89)	3.58 (0.87)	3.86 (0.91)
Q6: I have the skills I need to evaluate the health resources I find on the internet	3.63 (0.90)	3.52 (0.87)	3.81 (0.92)
Q7: I can tell high-quality from low-quality health resources on the internet	3.67 (0.90)	3.55 (0.88)	3.86 (0.90)
Q8: I feel confident in using information from the internet to make health decisions	3.59 (0.90)	3.48 (0.88)	3.76 (0.92)
Total score	29.22 (6.68)	28.36 (6.48)	30.65 (6.75)

**Table 2:** Distribution of the variables in rural and urban respondents.

Variable		Rural (n=4510), n (%)	Urban (n=2720), n (%)	Chi-square (*df*)	*P* value
**eHealth literacy**				159.781 (1)	<.001
	≥32	1673 (37.10)	1422 (52.28)		
	<32	2837 (62.90)	1298 (47.72)		
**Gender**				58.356 (1)	<.001
	Male	1355 (30.04)	1055 (38.79)		
	Female	3155 (69.96)	1665 (61.21)		
**Religious belief**				18.783 (1)	<.001
	Yes	291 (6.45)	110 (4.04)		
	No	4219 (93.55)	2610 (95.96)		
**Class ranking**				9.832 (3)	.02
	<25.0%	1217 (26.98)	781 (28.71)		
	25.0%-<50.0%	1623 (35.99)	1026 (37.72)		
	50.0%-<75.0%	1233 (27.34)	658 (24.19)		
	≥75.0%	437 (9.69)	255 (9.38)		
**Monthly per capita household income (RMB^a^)**				10002.180 (3)	<.001
	<2500	1158 (25.68)	219 (8.05)		
	2500-<5000	2163 (47.96)	852 (31.32)		
	5000-<10,000	952 (21.11)	1030 (37.87)		
	≥10,000	237 (5.25)	619 (22.76)		
**Exercise**				28.080 (1)	<.001
	Yes	3078 (68.25)	2016 (74.12)		
	No	1432 (31.75)	704 (25.88)		
**Smoking**				0.008 (1)	.93
	Yes	141 (3.13)	84 (3.09)		
	No	4369 (96.87)	2636 (96.91)		
**Drinking**				49.534 (1)	<.001
	Yes	626 (13.88)	549 (20.18)		
	No	3884 (86.12)	2171 (79.82)		
**7-item Generalized Anxiety Disorder**				8.135 (1)	.002
	≥5	1812 (40.18)	1001 (36.80)		
	<5	2698 (59.82)	1719 (63.20)		
**9-item Patient Health Questionnaire**				4.420 (1)	.04
	≥5	2141 (47.47)	1222 (44.93)		
	<5	2369 (52.53)	1498 (55.07)		

^a^1 RMB=US $0.14.

### Comparison of Variables’ Distribution

Table 3 presents the relationship between covariate distribution and eHealth literacy qualification status among urban and rural university students. The data indicate that certain covariate distributions share similar characteristics among students who passed or failed the eHealth literacy assessment. Gender, religious belief, monthly per capita household income, and drinking were identified as factors associated with eHealth literacy among university students.

**Table 3:** Distribution of the variables in unqualified eHealth literacy and qualified eHealth literacy respondents.

Variable			Unqualified eHealth literacy									Qualified eHealth literacy								
		Rural (n=2837), n (%)		Urban (n=1298), n (%)		Chi-square (*df*)		*P* value		Rural (n=1673), n (%)			Urban (n=1422), n (%)		Chi-square (*df*)		*P* value			
**Gender**							30.794 (1)		<.001							19.263 (1)		<.001		
	Male	807 (28.45)		481 (37.06)						548 (32.76)			574 (40.37)							
	Female	2030 (71.55)		817 (62.94)						1125 (67.24)			848 (59.63)							
**Religious belief**							15.514 (1)		<.001							4.876 (1)		.03		
	Yes	184 (6.49)		45 (3.47)						107 (6.40)			65 (4.57)							
	No	2653 (93.51)		1253 (96.53)						1566 (93.60)			1357 (95.43)							
**Class ranking**							3.912 (3)		.27							5.043 (3)		.17		
	<25.0%	713 (25.13)		336 (25.89)						504 (30.13)			445 (31.29)							
	25.0%-<50.0%	1032 (36.38)		487 (37.52)						591 (35.33)			539 (37.90)							
	50.0%-<75.0%	802 (28.27)		330 (25.42)						431 (25.76)			328 (23.07)							
	≥75.0%	290 (10.22)		145 (11.17)						147 (8.79)			110 (7.74)							
**Monthly per capita household income (RMB^a^)**							536.789 (3)		<.001							389.931 (3)		<.001		
	<2500	789 (27.81)		117 (9.01)						369 (22.06)			102 (7.17)							
	2500-5000	1406 (49.56)		458 (35.29)						757 (45.25)			394 (27.71)							
	5000-10,000	523 (18.43)		465 (35.82)						429 (25.64)			565 (39.73)							
	≥10,000	119 (4.19)		258 (19.88)						118 (7.05)			361 (25.39)							
**Exercise**							2.261 (1)		.13							0.791 (1)		.37		
	Yes	1652 (58.23)		788 (60.71)						1426 (85.24)			1228 (86.36)							
	No	1185 (41.77)		510 (39.29)						247 (14.76)			194 (13.64)							
**Smoking**							0.092 (1)		.76							0.298 (1)		.59		
	Yes	97 (3.42)		42 (3.24)						44 (2.63)			42 (2.95)							
	No	2740 (96.58)		1256 (96.76)						1629 (97.37)			1380 (97.05)							
**Drinking**							31.217 (1)		<.001							18.871 (1)		<.001		
	Yes	395 (13.92)		270 (20.80)						231 (13.81)			279 (19.62)							
	No	2442 (86.08)		1028 (79.20)						1442 (86.19)			1143 (80.38)							
**7-item Generalized Anxiety Disorder**							0.612 (1)		.43							0.572 (1)		.45		
	≥5	1285 (45.29)		571 (43.99)						527 (31.50)			430 (30.24)							
	<5	1552 (54.71)		727 (56.01)						1146 (68.50)			992 (69.76)							
**9-item Patient Health Questionnaire**							0.002 (1)		.97							0.002 (1)		.97		
	≥5	1528 (53.86)		700 (53.93)						613 (36.64)			522 (36.71)							
	<5	1309 (46.14)		598 (46.07)						1060 (63.36)			900 (63.29)							

^a^1 RMB=US $0.14.

### Logistic Model Results

Table 4 presents the results of logistic modeling for eHealth literacy qualification among urban and rural university students. Among rural university students, the following were identified as risk factors for failing to meet the eHealth literacy standard: female gender (odds ratio [OR] 0.847), class ranking in the 25.0%-49.9% and 50.0%-74.9% ranges (both OR 0.788), a PHQ-9 score of ≤5 (OR 0.648), and lack of exercise (OR 0.260). Conversely, higher monthly per capita household income was identified as a protective factor (5000-10,000 RMB: OR 1.607; ≥10,000 RMB: OR 1.944). Among urban university students, risk factors included class ranking in the top 25% (OR 0.708), a PHQ-9 score of <5 (OR 0.603), and lack of exercise (OR 0.267).

In conclusion, the discrepancies in eHealth literacy between urban and rural university students were primarily reflected in 2 key areas. First, gender was a significant factor only in rural settings, where being female was associated with a lower likelihood of meeting the eHealth literacy standard (OR 0.847). Second, higher monthly per capita household income (5000-10,000 RMB: OR 1.607; ≥10,000 RMB: OR 1.944) served as a protective factor in rural contexts but was not significant in urban ones.

**Table 4:** Logistic regression results for sociodemographic characteristics associated with eHealth literacy levels.

Variable		Overall, odds ratio (95% CI)	Rural, odds ratio (95% CI)	Urban, odds ratio (95% CI)
**Gender**				
	Male	Reference	Reference	Reference
	Female	0.843^a^ (0.755-0.940)	0.847^a^ (0.733-0.980)	0.893 (0.754-1.058)
**Religious belief**				
	Yes	Reference	Reference	Reference
	No	0.935 (0.752-1.163)	1.003 (0.772-1.303)	0.688 (0.454-1.042)
**Class ranking**				
	<25.0%	Reference	Reference	Reference
	25.0%-<50.0%	0.816^a^ (0.720-0.924)	0.788^a^ (0.670-0.926)	0.864 (0.708-1.054)
	50.0%-<75.0%	0.798^a^ (0.696-0.915)	0.788^a^ (0.662-0.938)	0.830 (0.664-1.037)
	≥75.0%	0.791^a^ (0.653-0.958)	0.839 (0.655-1.074)	0.708^a^ (0.521-0.962)
**Monthly per capita household income (RMB^b^)**				
	<2500	Reference	Reference	Reference
	2500-5000	1.081 (0.939-1.245)	1.061 (0.904-1.246)	0.861 (0.626-1.184)
	5000-10,000	1.712^c^ (1.472-1.992)	1.607^c^ (1.331-1.940)	1.161 (0.848-1.589)
	≥10,000	2.079^c^ (1.727-2.503)	1.944^c^ (1.439-2.626)	1.273 (0.914-1.774)
**Exercise**				
	Yes	Reference	Reference	Reference
	No	0.262^c^ (0.232-0.296)	0.260^c^ (0.222-0.304)	0.267^c^ (0.220-0.324)
**Smoking**				
	Yes	Reference	Reference	Reference
	No	0.828 (0.608-1.128)	0.802 (0.532-1.209)	0.897 (0.554-1.452)
**Drinking**				
	Yes	Reference	Reference	Reference
	No	1.041 (0.903-1.200)	1.007 (0.826-1.228)	1.015 (0.826-1.249)
**7-item Generalized Anxiety Disorder**				
	≥5	Reference	Reference	Reference
	<5	0.867 (0.748-1.003)	0.839 (0.694-1.013)	0.920 (0.727-1.165)
**9-item Patient Health Questionnaire**				
	≥5	Reference	Reference	Reference
	<5	0.638^c^ (0.553-0.736)	0.648^c^ (0.539-0.780)	0.603^c^ (0.480-0.758)

^a^Significant at *P*<.05.

^b^1 RMB=US $0.14.

^c^Significant at *P*<.01.

### Decomposition Analysis Results

To ensure the stability of the results, the decomposition model was repeated 100 times using the Stata MP 18.0 (StataCorp). Multimedia Appendix 1 presents the decomposition model results for differences in eHealth literacy levels between urban and rural university students. The findings indicate that observed factors accounted for 28.6% of the disparity, while 71.4% was attributed to urban-rural differences and unobserved factors. Significant contributors to the eHealth literacy gap (*P*<.001) included monthly per capita household income (13.4%; *P*=.007), exercise habits (11.7%; *P*<.001), and PHQ-9 scores (2.1%; *P*<.001).

## Discussion

### Principal Findings

This study explores the relationship between eHealth literacy among urban and rural university students in China and factors such as sociodemographic characteristics, personal lifestyle, and psychological health. It quantifies the extent to which these factors contribute to disparities in eHealth literacy between the 2 groups. Our findings confirm significant differences in eHealth literacy levels between urban and rural students, influenced by multiple factors.

The study found that 57.2% of university students had eHealth literacy below the passing threshold, with an average score of 29.22 (6.68). These scores were comparable to those reported for Chinese university students (mean 30.16, SD 6.31) [34], higher than those of students from other Asian countries [49,50], and similar to those of their European and American peers [51,52]. However, all average scores remained below the qualifying standard, highlighting a concerning issue: overall eHealth literacy levels among university students are insufficient. Previous research by Bailey et al [53], Chesser et al [54], Gustafson et al [55], Mengestie et al [56], and others have documented disparities in eHealth literacy between urban and rural populations. Our study found that a significantly higher proportion of rural university students (2837/4510, 62.90%) did not meet eHealth literacy standards compared with their urban counterparts (1298/2720, 47.72%; *P*<.001). These findings highlight substantial urban-rural disparities in eHealth literacy among Chinese university students, aligning with previous research by Chinese scholars [57]. The study identified a 15.2% point gap between urban and rural students failing to meet eHealth literacy standards, emphasizing the critical role of the upbringing environment in shaping eHealth literacy. Enhancing eHealth literacy among rural students should be a priority to address and potentially mitigate these disparities. Notably, the study by Giger et al [58] found that eHEALS scores were not influenced by rural status or gender. This outcome may stem from uncertainties in the theoretical framework of eHealth literacy, potentially leading to misinterpretations of data measured by the eHEALS scale. Additionally, the lack of variation in scores could be due to a somewhat biased study population.

The logistic regression analysis revealed both similarities and differences in the covariates influencing eHealth literacy among Chinese urban and rural university students. Class ranking, exercise, and PHQ-9 scores were significant factors in both groups. Notably, better academic performance emerged as a protective factor for higher eHealth literacy. To some extent, higher grades indicate better information access and comprehension skills, aligning with findings from previous studies [12,23]. Exercise is a common protective factor, consistent with findings from Tsukahara et al [49], Mitsutake et al [21], and others. Exercise habits reflect an individual’s greater health awareness and willingness to seek information. Additionally, many physical activities facilitate social interaction and information sharing. Individuals with high eHealth literacy are also more likely to engage in regular exercise [59], which collectively contributes to the accumulation and enhancement of health literacy. Conversely, PHQ-9 scores were identified as a risk factor for eHealth literacy among both urban and rural students, a finding supported by previous studies [59-61]. Extensive research has confirmed that depressive symptoms negatively impact cognitive function [62], information processing, and health decision-making. Furthermore, depression directly undermines self-efficacy [63], which in turn influences health literacy both directly and indirectly [64], ultimately hindering its development and maintenance.

The covariates influencing eHealth literacy levels differ between urban and rural students. This study found that gender significantly affects rural students, with rural female students exhibiting lower eHealth literacy levels. The existing literature presents mixed findings on this issue. In countries with prevalent gender inequalities, women tend to have lower health literacy, whereas in some Western nations, women demonstrate higher health literacy levels [65,66]. In the context of this study, these results may partially reflect the persistent gender gap in education in rural China, as well as traditional societal views on gender roles. This disparity objectively limits rural female students’ access to and use of health information, while also indirectly influencing their health awareness, needs, and confidence in using eHealth resources. Additionally, potential gender differences in health needs, attitudes, and communication patterns underscore the importance of tailored interventions and support. By contrast, urban students do not exhibit significant gender differences, likely due to relatively greater resource availability and a more equitable social environment, where information dissemination is more diverse and access to electronic tools and health information is more convenient. In addition, monthly per capita household income serves as a protective factor for eHealth literacy among rural students but not among urban students. This may be because high-income rural families can better support students in accessing and utilizing eHealth information resources, whereas in urban settings, the impact of income differences is mitigated by greater environmental support and resource availability. The study [67] highlights the importance of tailoring eHealth literacy interventions to specific populations.

The results of the Fairlie decomposition model indicate that 28.6% of the difference in eHealth literacy between urban and rural students can be attributed to observed factors, primarily monthly per capita family income (13.4%), exercise habits (11.7%), and PHQ-9 scores (2.1%). These factors not only demonstrate statistical significance in the empirical data but also have clear, real-life implications, which is common in logistic regression analyses. Several studies have shown that family income is a key determinant of health literacy, both in the general population and among students [68], as it directly influences their ability to access health information. Students from higher-income families are more likely to afford smart devices and internet services, facilitating easier access to online medical resources [69]. Exercise, by contrast, reflects students’ healthy habits, which are not only positively correlated with health literacy but also subconsciously motivate them to seek more health knowledge [70]. Additionally, the PHQ-9 score, as a self-report measure of depressive states, suggests that students with higher scores may be less likely to engage with and comprehend health information due to low mood, a relationship that has been confirmed in studies on mental health and health literacy [71].

In this study, 71.4% of the difference in eHealth literacy between urban and rural students was attributed to unobserved factors, which may be linked to structural disparities between urban and rural areas. Health inequalities between urban and rural populations remain a global issue, with developing countries experiencing more pronounced disparities than developed countries [72]. Strengthening health services in rural communities is widely recognized as a priority. Multiple factors contribute to the observed disparities in health outcomes between rural and urban areas [73]. The pervasive socioeconomic disadvantages endemic to rural areas often result in reduced access to health care services, perpetuating a cycle of disadvantage. Geographic isolation and a shortage of health care providers further exacerbate this issue. Additionally, rural populations face higher risks of injury, encounter significant barriers related to transportation and communication, and are affected by diseconomies of scale due to their sparse distribution. Collectively, these factors negatively impact the health outcomes of individuals living in nonmetropolitan areas. Nevertheless, evidence suggests that even after geographic barriers are removed, health service utilization remains low in some rural communities [74]. Additionally, studies indicate that barriers to self-management—such as limited formal education and poverty—may be further exacerbated in areas with fewer health care professionals and weaker health infrastructure [75]. Patterson et al [76] found that adjusting for socioeconomic status did not fully account for the observed differences in health risk factors between rural and urban populations. Moreover, other factors that differ between these regions may further contribute to disparities in health outcomes. Given the critical role of eHealth literacy in health outcomes, it is plausible that it represents one of these contributing factors.

Existing research has confirmed the crucial role of mobile technology in enhancing eHealth literacy and improving health outcomes, particularly in rural areas and among disadvantaged groups [77]. As health services continue to transition to digital platforms, the need to promote eHealth literacy becomes increasingly urgent. Our findings offer valuable insights for policy makers. First, efforts should be made to improve access to eHealth resources in rural and underserved areas. Additionally, the government should implement measures to enhance residents’ incomes and living conditions, thereby addressing structural barriers to eHealth engagement. Given the urban-rural divide, collaboration between the government and educational institutions is essential to developing a multichannel eHealth education platform. This platform should provide tailored eHealth knowledge and telemedicine services, such as live-streamed health lectures and regular dissemination of health information, specifically targeting rural and resource-limited areas to bridge the urban-rural information access gap. Importantly, interventions should be designed with consideration for less educated and lower-income populations, who have the greatest need for such support [78]. Second, fostering a positive environment for physical activity can help encourage the adoption of healthy behaviors, as health literacy and healthy behaviors are mutually reinforcing [79]. To encourage students to develop healthy exercise habits, universities should collaborate with local communities to establish well-equipped sports facilities and organize regular sports events and health-related activities. Additionally, implementing a reward system—where students earn points for participating in sports and health lectures, redeemable for incentives—could further motivate engagement. Third, expanding on-campus mental health services is crucial for enhancing students’ ability to process health information. Given the significant impact of mental health on eHealth literacy observed in this study, universities should establish a comprehensive mental health support system. This could include online psychological counseling, crisis intervention services, and routine psychological assessments to ensure early detection and support. Universities should provide high-quality online mental health resources through their health and counseling center websites [80] and establish an integrated framework linking mental health support with eHealth literacy training. This approach would enhance students’ understanding and application of health knowledge while effectively reducing barriers to accessing health information. Finally, eHealth literacy training should be systematic, with a structured approach to evaluating its effectiveness. The influence of education on health is well established [81], and numerous studies have shown that elective courses can significantly impact students’ health literacy levels [82,83]. Universities can implement targeted eHealth literacy training programs that comprehensively cover all aspects of eHealth literacy. Regular monitoring through surveys and evaluations can help identify educational gaps and optimize the curriculum accordingly. Additionally, fostering supportive environments at home and school can enhance students’ self-efficacy and confidence, ultimately contributing to improved health outcomes.

### Strengths and Limitations

This study is the first to investigate disparities in eHealth literacy between urban and rural university students in China. Using Fairlie’s model for quantitative decomposition, we identified key influencing factors and their specific contributions. However, certain limitations must be acknowledged. First, as this study is based on a cross-sectional questionnaire survey, it does not capture dynamic changes in participants’ eHealth literacy over time, limiting our ability to establish causal relationships between eHealth literacy and urban-rural factors. Second, although the eHEALS has been widely validated and is considered reliable across various cultural contexts and countries, it remains a self-assessment tool and is therefore susceptible to individual subjective biases. Notably, large-scale epidemiological studies worldwide, such as the UK Biobank and China Health and Retirement Longitudinal Study (CHARLS) databases, often rely on self-reported data. These subjective assessments can be supplemented with objective indicators to provide a more comprehensive evaluation of actual eHealth skill levels. Third, our sample size is relatively limited compared with the overall population of university students. Therefore, caution should be exercised when generalizing the findings to a broader or global scale. To enhance the universality of these results, future studies could refine the sampling method and expand the survey scope. Comparative studies in other countries could also provide insights into the similarities and differences between China and other contexts. Finally, while this study considered a broad range of demographic, sociological, and personal lifestyle factors, eHealth literacy is influenced by numerous variables. Future research should explore additional influencing factors. Moreover, if disparities in eHealth literacy between urban and rural students continue to be observed, it will be crucial to develop scientifically sound, context-specific interventions. Researchers should also focus on validating, refining, and addressing potential shortcomings in the implementation of these interventions.

### Conclusions

The regression and decomposition analyses in this study revealed that urban university students had higher eHealth literacy levels than their rural counterparts. Key factors contributing to this disparity included monthly per capita household income, exercise habits, and PHQ-9 scores. The Fairlie decomposition model indicated that 28.6% of the difference in eHealth literacy between urban and rural students could be attributed to observed factors, with monthly per capita household income accounting for 13.4%, exercise for 11.7%, and PHQ-9 scores for 2.1%. These findings provide novel insights into urban-rural disparities in eHealth literacy among Chinese university students, offering valuable guidance for developing or refining policies to enhance eHealth literacy.

The policy strategies specifically include the following: (1) improving access to eHealth resources in rural and underserved areas; (2) fostering an environment that encourages physical activity to promote healthy behaviors; (3) expanding school-based mental health services to enhance health information processing capacity; and (4) implementing systematic eHealth literacy training with ongoing evaluation. These strategies and policies will help promote equitable access to and utilization of eHealth resources for all students, regardless of their location.

## Appendix

Multimedia Appendix 1 The Fairlie decomposition Model of eHealth literacy Status in Urban and Rural university students.

## Abbreviations

CHARLS: China Health and Retirement Longitudinal Study
eHEALS: eHealth Literacy Scale
FDA: Fairlie decomposition analysis
GAD-7: 7-item Generalized Anxiety Disorder
OR: odds ratio
PHQ-9: 9-item Patient Health Questionnaire
