# Supplementary material for: Decomposition and Comparative Analysis of Urban-Rural Disparities in eHealth Literacy Among Chinese University Students: Cross-Sectional Study
Source: J Med Internet Res. 2025 Mar 26;27:e63671. doi: 10.2196/63671 (PMC11982776; doi:10.2196/63671)
Supplement: Multimedia Appendix 1 [file jmir_v27i1e63671_app1.docx]

*Table 5.*The Fairlie decomposition Model of eHealth literacy Status in Urban and Rural university students

| **Terms of decomposition** | | | **eHealth literacy status** | |
| --- | --- | --- | --- | --- |
| Difference | | | 0.152 | |
| Explained (%) | | | 0.043(28.6) | |
| Non-explained (%) | | | 0.108(71.4) | |
| **Contribution to differences** | ***β*** | ***P*** | **Contribution (%)** | **[95%CI]** |
| **Explained** |  |  |  |  |
| Monthly per capita household income | 0.020 | 0.007 | 13.4 | (0.006,0.035) |
| Exercise | 0.018 | ＜.001 | 11.7 | (0.015,0.020) |
| PHQ-9 | 0.003 | ＜.001 | 2.1 | (0.002,0.005) |
| Gender | 0.002 | 0.190 | 1.4 | (-0.001,0.005) |
| Class ranking | 0.001 | 0.240 | 0.6 | (-0.001,0.002) |
| GAD-7 | 0.001 | 0.490 | 0.5 | (-0.001,0.003) |
| Drinking | 0.0002 | 0.890 | 0.1 | (-0.003，0.003) |
| Smoking | -0.00002 | 0.860 | -0.01 | (-0.0003，0.0002) |
| Religious belief | -0.002 | 0.060 | -1.2 | (-0.001,0.00006) |
